# The Key Genes Underlying Pathophysiology Association between Plaque Instability and Progression of Myocardial Infarction

**DOI:** 10.1155/2021/4300406

**Published:** 2021-12-09

**Authors:** Yue Zheng, Yijie Gong, Yuheng Lang, Zhenchang Qi, Xiaomin Hu, Tong Li

**Affiliations:** ^1^School of Medicine, Nankai University, Tianjin 300071, China; ^2^Department of Cardiac Center, The Third Central Hospital of Tianjin, 83 Jintang Road, Hedong District, Tianjin 300170, China; ^3^The Third Central Clinical College of Tianjin Medical University, Tianjin 300170, China; ^4^The Third Central Hospital of Tianjin, 83 Jintang Road, Hedong District, Tianjin 300170, China; ^5^Tianjin Key Laboratory of Extracorporeal Life Support for Critical Diseases, Tianjin, China; ^6^Institute of Hepatobiliary Disease, Tianjin, China

## Abstract

Young patients with type 2 diabetes and myocardial infarction (MI) have higher long-term all-cause and cardiovascular mortality. In addition, the observed increased, mildly abnormal baseline lipid levels, but not lipid variability, are associated with an increased risk of atherosclerotic cardiovascular disease events, particularly MI. This study investigated differentially expressed genes (DEGs), which might be potential targets for young patients with MI and a high-fat diet (HFD). GSE114695 and GSE69187 were downloaded and processed using the limma package. A Venn diagram was applied to identify the same DEGs, and further pathway analysis was performed using Metascape. Protein-protein interaction (PPI) network analysis was then applied, and the hub genes were screened out. Pivotal miRNAs were predicted and validated using the miRNA dataset in GSE114695. To investigate the cardiac function of the screened genes, an MI mouse model, echocardiogram, and ELISA of hub genes were applied, and a correlation analysis was also performed. From aged mice fed HFD, 138 DEGs were extracted. From aged mice fed with chow, 227 DEGs were extracted. Pathway enrichment analysis revealed that DEGs in aging mice fed HFD were enriched in lipid transport and lipid biosynthetic process 1 d after MI and in the MAPK signaling pathway at 1 w after MI, suggesting that HFD has less effect on aging with MI. A total of 148 DEGs were extracted from the intersection between plaques fed with HFD and chow in young mice and MI_1d, respectively, which demonstrated increased inflammatory and adaptive immune responses, in addition to myeloid leukocyte activation. A total of 183 DEGs were screened out between plaques fed with HFD vs. chow in young mice and MI_1w, respectively, which were mainly enriched in inflammatory response, cytokine production, and myeloid leukocyte activation. After validation, PAK3, CD44, CD5, SOCS3, VAV1, and PIK3CD were demonstrated to be negatively correlated with LVEF; however, P2RY1 was demonstrated to be positively correlated. This study demonstrated that the screened hub genes may be therapeutic targets for treating STEMI patients and preventing MI recurrence, especially in young MI patients with HFD or diabetes.

## 1. Introduction

Acute myocardial infarction (MI) remains one of the leading causes of death in patients [1]. In addition, myocardial infarction mortality has increased 5.6-fold over the past 30 years [2]. Young patients with type 2 diabetes and MI have higher long-term all-cause and cardiovascular mortality, and more than one-third of patients die within 10 years, which indicates that more aggressive secondary prevention is needed for these patients [3]. Mildly abnormal baseline lipid levels are associated with an increased risk of atherosclerotic cardiovascular disease events, particularly MI, whereas lipid variability is not [3, 4]. In these patients, early diagnosis of coronary disease progression can significantly reduce mortality and thus save lives [5].

Previous studies have reported that acute myocardial infarction promotes the release of progenitor cells and hematopoietic stem cells from the bone marrow niche. These progenitor cells subsequently colonize the spleen, thereby increasing the number of monocytes in the blood, which further promotes atherosclerosis and thus myocardial infarction progression [6, 7]. Endothelial vasomotor function in the coronary arteries of patients with ST-segment elevation myocardial infarction (STEMI) is consistently impaired and is strongly associated with atherosclerosis development and plaque progression [8]. Therefore, biomarkers for plaque progression in patients may become new therapeutic targets to prevent coronary disease progression and MI recurrence and may be useful for predicting MI morbidity in young patients and decreasing mortality.

Recently, more genetic data related to myocardial infarction have been obtained through gene microarrays of patient peripheral blood and mouse myocardium as well as RNA sequencing assays [9–11]. However, bioinformatic analysis has rarely been used for data mining in cardiovascular diseases, especially for coronary progression and myocardial infarction recurrence. In this study, two GEO datasets, GSE69187 and GSE114695, were downloaded, and further analysis was conducted to identify the hub genes, which will help diagnose the progression of coronary heart disease at an early stage and reduce the mortality rate.

## 2. Materials and Methods

### 2.1. Microarray Data

Using the keywords “atherosclerosis” or “myocardial infarction,” two GEO datasets were screened: GSE114695 contributed by Park et al. and GSE69187 contributed by Du et al. Aorta microarray data of young and aged LDLr-deficient mice fed with chow or a high-fat diet (HFD) was found in GSE69187. Left ventricle microarray data, including mRNA and miRNA microarray data, at 1 d or 1 or 8 wk subsequent to MI was found in GSE114695. Volcano plots were plotted using GraphPad Prism 7.0.

### 2.2. Data Processing

The original expression matrix was normalized and processed using R. The limma package was used to screen differentially expressed genes (DEGs). The *P* value of genes was calculated using Student's *t*-test, and the adjusted *P* value was calculated using Benjamini and Hochberg's method. A log2(fold change) (FC) > 1 and adjusted *P* value < 0.05 were applied as the cut-off criteria.

### 2.3. Enrichment Analyses

Venn diagrams were applied to identify the same DEGs, and subsequent GO and KEGG pathway analyses were performed using Metascape (https://metascape.org/gp/index.html#/main/step1). Statistical significance was defined to be *P* < 0.05.

### 2.4. Gene Cluster Identification and Protein-Protein Interaction (PPI) Network Analysis

To investigate the hub genes, DEGs were uploaded to STRING (version 11) to obtain the protein network interaction diagram. The results of STRING analysis were imported into Cytoscape v.3.7.1, and the hub genes were investigated by applying the Cytoscape plug-in (MCODE). The screened gene cluster was then uploaded to NetworkAnalyst 3.0 (https://www.networkanalyst.ca/NetworkAnalyst/home.xhtml) for further verification. A DEG degree > 30 can be used for further prediction of pivotal miRNAs.

### 2.5. Prediction of Pivotal miRNAs and Construction of Gene-miRNA Interaction Network Analysis

Using miRWalk 2.0, DEG-targeted miRNAs were predicted. To verify the accuracy of the results, the miRNA dataset GSE114695 and predicted miRNAs were used to perform intersections. The final result obtained from the intersection was further processed using Cytoscape v 3.7.1.

### 2.6. MI Model Construction

Adult experimental C57Bl/6J male mice were purchased from Charles River (Beijing, China). Mice were maintained in a specific pathogen-free environment (temperature: 23-25°C; humidity: 55-60%) with free access to food and water and a 12/12 light-dark cycle. Protocols were approved by the Institute of Radiation Medicine, Chinese Academy of Medical Science, which conform to the *Guide for the Care and Use of Laboratory Animals* published by the US National Institutes of Health (8th edition, 2011).

The mice were fed HFD 4 wk after birth (12–15 g). Fed with HFD for 2 months (Western diet, HFHC100244), MI was induced in young adults (*n* = 6 per time point in each group, 28-32 g, 10–11 wk). Briefly, the mice were exposed to isoflurane (1.5–2%, MSS-3, England), and the left coronary artery was located, sutured, and ligated at a site approximately 3 mm from its origin, which induced approximately 50% ischemia in the left ventricle in mice. Infarction was considered successful by visually confirming pale discoloration and ST elevation on the electrocardiogram. Sham-operated animals underwent the same procedure as the MI model without any coronary artery ligation, and mice were fed HFD after the surgery, till they were euthanized by cervical dislocation after administering isoflurane (5%, MSS-3, England). Samples from the left ventricle were collected for further analysis.

### 2.7. Echocardiographic Examination

Cardiac function was evaluated using a Vevo 2100 System equipped with a 30 MHz transducer (FUJIFILM VisualSonics, Inc. Toronto, Canada) 1 d and 1 and 8 wk subsequent to the surgery. The investigator was blinded to group assignment. Mice were anesthetized by administering isoflurane (1–1.5%, MSS-3, England) and moved to a warming plate that maintained the core body temperature. Heart function was detected through a two-dimensional parasternal long axis. The limb lead electrocardiogram (ECG) was also recorded, and the corresponding PR and QRS intervals of each group were measured and analyzed based on the ECG records of at least 100 beats. Left ventricular ejection fraction (LVEF, %) and fractional shortening (LVFS, %) were measured using the M-mode.

### 2.8. Enzyme-Linked Immunosorbent Assay

The infarcted area and border zone (500 *μ*g) were collected, and the proteins were extracted according to the instructions in the kit (BC3710, Solarbio). For further validation, the left ventricular sample levels, including IQGAP2, Pak3, Slit2, CD44, CD5, SOCS3, and P2RY1, were measured using a mouse IQGAP2 ELISA kit (ybE288Mu, Ambion, China), mouse Pak3 ELISA kit (CSB-PA017407LA01HU, CUSABIO), mouse Slit2 ELISA kit (Q9R1B9, RayBiotech), mouse CD44 ELISA kit (P15379, RayBiotech), mouse CD5 ELISA kit (Q91X69, RayBiotech), mouse SOCS3 ELISA kit (EKU07517, BIOMATIK), mouse Vav1 ELISA kit (JN-S-86921, AFZHAN), and mouse P2RY1 ELISA kit (CSB-EL017326MO, CUSABIO), respectively.

Samples from the left ventricle in the border zone and infarcted area were incubated with primary antibodies overnight at 4°C and then incubated with secondary antibodies for 1 h at room temperature. Antibodies against RASD2 (CUSABIO) and PIK3CD (CAF13567, BIOMATIK) were used.

### 2.9. Statistical Analysis

All data are presented as the mean ± SD. Statistical analyses were performed using SPSS 23.0. The Shapiro-Wilk normality test and Welch *t*-test (two groups) were used, and the Spearman correlation analysis was used to measure the LVEF, LVFS, and protein levels of screened hub genes. Statistical significance was defined to be *P* < 0.05.

## 3. Results and Discussion

### 3.1. Identification of DEGs in GSE114695 and Enrichment Analysis

**Figure 1:**
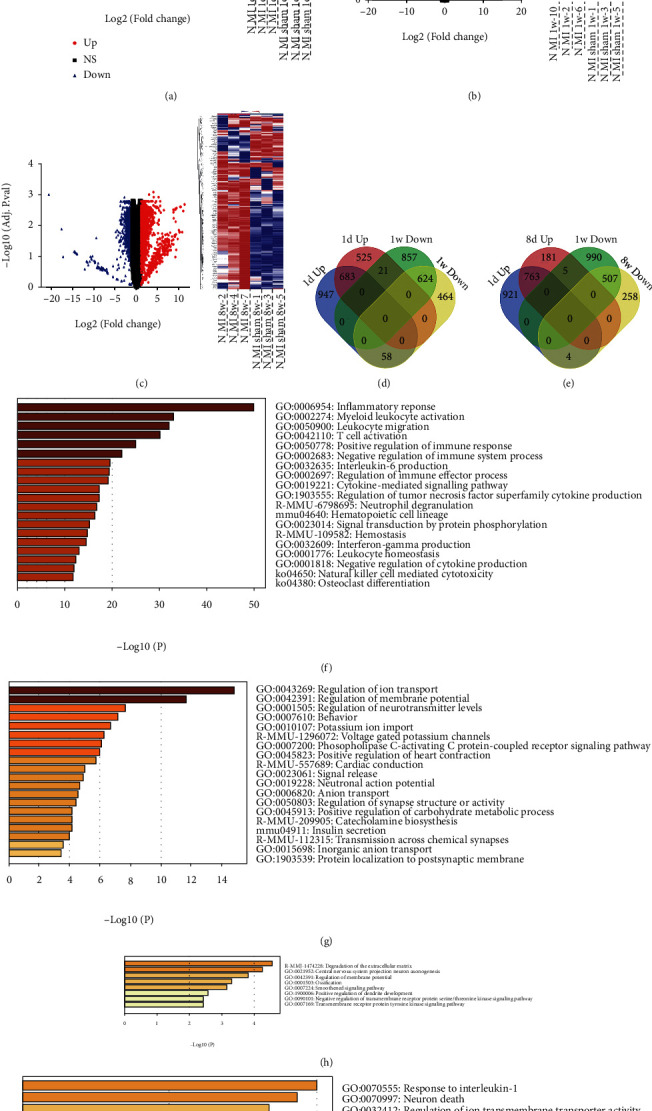
Identification of DEGs in GSE114695 and enrichment analysis. (a–c) Volcano plot and heat map of 1 d (a), 1 w (b), and 8 w (c) after MI in GSE114695. (d) Venn diagram of upregulated and downregulated DEGs at 1 d and 1 w after MI. (e) Venn diagram of upregulated and downregulated DEGs at 1 w and 8 w after MI. (f) Enrichment analysis of DEGs which were both upregulated at 1 d and 1 w after MI. (g) Enrichment analysis of DEGs which were both downregulated at 1 d and 1 w after MI. (h) Enrichment analysis of DEGs which were downregulated at 1 d but upregulated at 1 w. (i) Enrichment analysis of DEGs which were upregulated at 1 d but downregulated at 1 w.

Using the limma package, 1462 upregulated and 1367 downregulated DEGs were obtained in the GSE114695 MI_1d dataset, 1879 upregulated and 1617 downregulated DEGs were obtained in the MI_1w dataset, and 1303 upregulated and 979 downregulated DEGs were obtained in the MI_8w dataset after log2 transformation (Figures 1(a)–1(c), Figure S1). The DEGs were also screened in GSE69187 after log2 transformation (Figure S2).

Using a Venn diagram, 683 DEGs were upregulated 1 d and 1 wk after the MI, and 624 DEGs were downregulated at 1 d and 1 wk after MI. However, 58 DEGs were downregulated after 1 d but upregulated after 1 wk, and 21 DEGs were upregulated after 1 d but downregulated after 1 wk (Figure 1(d)). Enrichment analysis by Metascape showed that 683 DEGs were mainly enriched in the inflammatory response, myeloid leukocyte activation, and T cell activation, whereas 624 DEGs were mainly enriched in the regulation of ion transport, membrane potential, and neurotransmitter levels. In addition, 58 DEGs were mainly enriched in the degradation of the extracellular matrix, and 21 DEGs were mainly enriched in response to interleukin-1 and neuronal death (Figures 1(f)–1(i); Tables S1 and S2).

Using a Venn diagram, 763 DEGs were upregulated 1 and 8 wk after MI, and 507 DEGs were downregulated at 1 and 8 wk after MI. However, nine DEGs differed between 1 and 8 wk (Figure 1(e)). Enrichment analysis demonstrated that 763 DEGs were mainly enriched in the inflammatory response, cell-cell adhesion, and extracellular matrix organization, while 507 DEGs were mainly enriched in the regulation of ion transmembrane transport, membrane potential, and action potential. In addition, nine DEGs were enriched in the positive regulation of cell morphogenesis involved in differentiation, glial cell differentiation, and regulation of neuron differentiation (Figure S3; Tables S3 and S4).

### 3.2. HFD Shows Less Effect on Aging with MI

**Figure 2:**
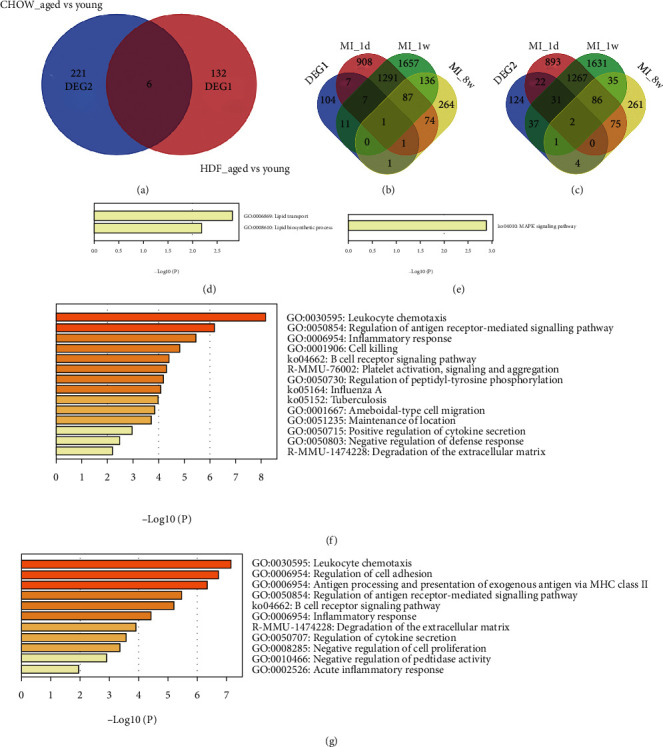
HFD shows less effect on aging with MI. (a) Venn diagram showed DEGs of plaque samples among aged mice or young mice fed with HFD or chow, and 132 DEGs and 221 DEGs were obtained named DEG1 and DEG2. (b) Venn diagram of DEG1 and DEGs at 1 d, 1 w, and 8 w in GSE114695. (c) Venn diagram of DEG2 and DEGs at 1 d, 1 w, and 8 w in GSE114695. (d) 16 DEGs were screened out between DEG1 and DEGs at 1 d, which were enriched in lipid transport and lipid biosynthetic process. (e) 19 DEGs were screened out between DEG1 and DEGs at 1 w, which were enriched in the MAPK signaling pathway. (f) 55 DEGs were screened out between DEG2 and DEGs at 1 d, which were enriched in leukocyte chemotaxis, inflammatory response, and cell killing. (g) 71 DEGs were screened out between DEG2 and DEGs at 1 w, which were mainly enriched in leukocyte chemotaxis and regulation of cell adhesion.

Compared to young mice fed HFD, 138 DEGs were extracted from aged mice fed HFD. Compared to young mice fed chow, 227 DEGs were extracted from aged mice fed chow. To determine the difference between the two lifestyles, a Venn diagram was used, and 132 DEGs and 221 DEGs were identified, named DEG1 and DEG2, respectively (Figure 2(a)). To further screen the DEGs related to MI, Venn diagrams were also utilized to obtain the same DEGs among MI microarray data, DEG1 and DEG2 (Figures 2(b) and 2(c)). Sixteen DEGs were identified between DEG1 and MI_1d, which were enriched in the lipid transport and lipid biosynthetic processes (Figure 2(d)). However, the other 2360 DEGs were demonstrated in an enhanced inflammatory response, leukocyte migration, regulation of cytokine production, and T cell activation (Figure S4). In addition, 19 DEGs were screened between DEG1 and MI_1w, which enhanced the MAPK signaling pathway (Figure 2(e)). The other 3171 DEGs were mainly enhanced in cell-cell adhesion, inflammatory response, and regulation of ion transport (Figure S5). Only three DEGs were observed between DEG1 and MI_8w.

In contrast, 55 DEGs were screened between DEG2 and MI_1d, which were enriched in leukocyte chemotaxis, inflammatory response, and cell killing (Figure 2(f)). In addition, 71 DEGs were screened out between DEG2 and MI_1w, which were mainly enriched in leukocyte chemotaxis and regulation of cell adhesion (Figure 2(g)). Seven DEGs were identified between DEG2 and MI_8w.

### 3.3. The HFD Effect on Aged Mice or Young Mice with MI

**Figure 3:**
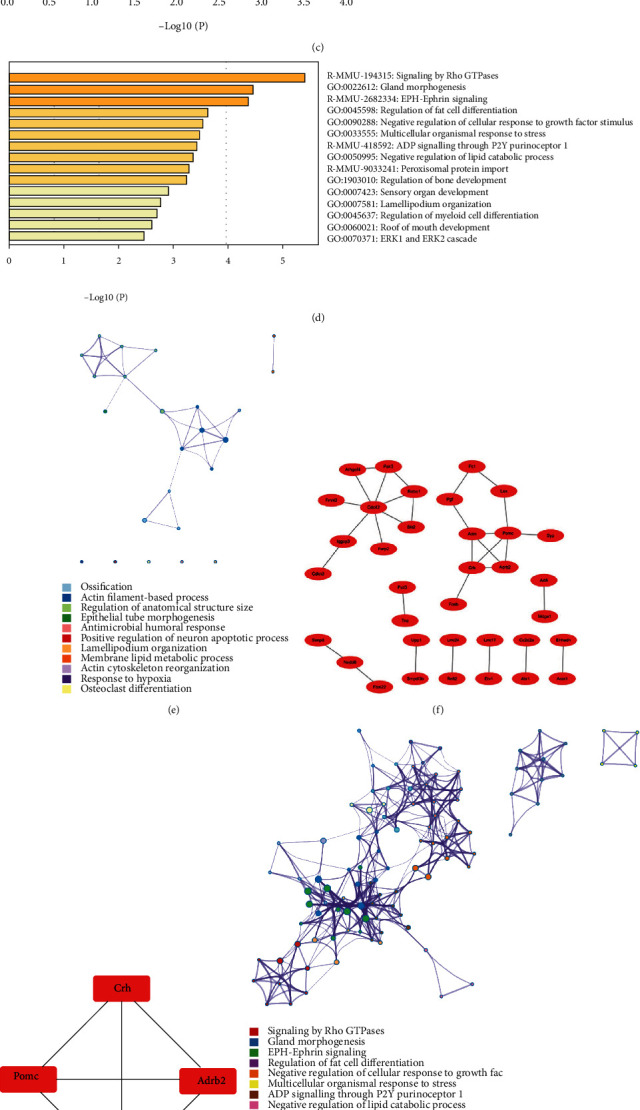
The HFD effect on aged mice with MI. (a) Venn diagram showed DEGs of plaque samples between aged mice and young mice fed with HFD and fed with chow, and 1263 DEGs and 623 DEGs were obtained named DEG1 and DEG2, respectively. (b) Venn diagram of DEG3 and DEGs at 1 d, 1 w, and 8 w in GSE114695. (c) Enrichment analysis of 83 DEGs was obtained from the intersection between DEG3 and DEGs at 1 d. (d) Enrichment analysis of 130 DEGs was obtained from the intersection between DEG3 and DEGs at 1 w. (e) Network of enriched pathways between DEG3 and DEGs at 1 d. (f, g) PPI network (f) and 1 module (g) were obtained between DEG3 and DEGs at 1 d. (h) Network of enriched pathways between DEG3 and DEGs at 1 w. (i, j) PPI network (i) and 4 modules (j) were obtained between DEG3 and DEGs at 1 w.

To investigate the effect of HFD on aged mice, the DEGs in aged mice fed with HFD and with chow were screened and compared; 1263 DEGs (DEG3) were obtained, and further Venn diagram analysis was used to determine the enriched pathways among DEG3 and MI microarray data (Figure 3(a)). Eighty-three DEGs were obtained from the intersection between DEG3 and MI_1d, mainly enriched in ossification, actin filament-based process, and regulation of anatomical structure size (Figures 3(b), 3(c), and 3(e); Table 1). Next, PPI network analysis was applied, and one module was obtained, including Crh, Adrb2, Pomc, and Adm (Figures 3(f) and 3(g)). In addition, 130 DEGs were screened between DEG3 and MI_1w, which were mainly enhanced in signaling by Rho GTPases, gland morphogenesis, EPH-Ephrin signaling, and regulation of fat cell differentiation (Figures 3(d) and 3(h); Table 2). Further PPI network analysis demonstrated that four modules were related to the pathways, such as E2f1, Uhrf1, Cdc20, AURKA, and Egfr (Figures 3(i) and 3(j)).

**Figure 4:**
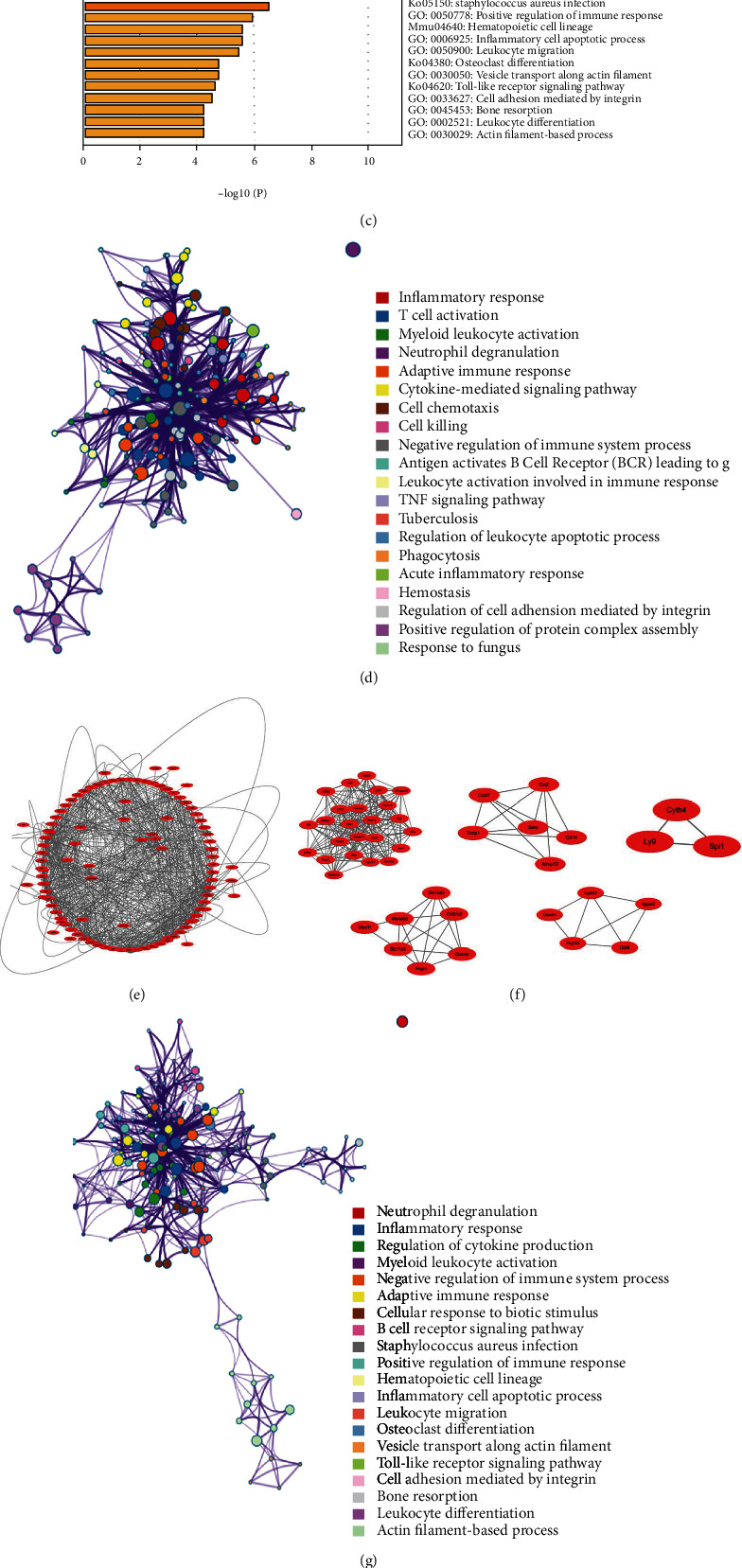
The HFD effect on young mice with MI. (a) Venn diagram of DEG4 and DEGs at 1 d, 1 w, and 8 w in GSE114695. (b) Enrichment analysis of 148 DEGs was obtained from the intersection between DEG4 and DEGs at 1 d. (c) Enrichment analysis of 183 DEGs was obtained from the intersection between DEG4 and DEGs at 1 w. (d) Network of enriched pathways between DEG4 and DEGs at 1 d. (e, f) PPI network (e) and 5 modules (f) were obtained between DEG4 and DEGs at 1 d. (g) Network of enriched pathways between DEG4 and DEGs at 1 w. (h, i) PPI network (h) and 6 modules (i) were obtained between DEG4 and DEGs at 1 w.

In contrast, to investigate the effect of HFD on young mice, DEGs in young mice fed HFD and chow were screened and compared; 623 DEGs (DEG4) were obtained, and a Venn diagram was also utilized to identify the enriched pathways among the DEG4 and MI microarray data (Figure 3(a)). A total of 148 DEGs were obtained from the intersection between DEG4 and MI_1d, which were mainly enriched in inflammatory response, T cell activation, adaptive immune response, and myeloid leukocyte activation (Figures 4(a), 4(b), and 4(d); Table 3). Next, PPI network analysis was applied, and five modules were related: Ccl2, Ly9, Cxcl1, Was, and Cd14 (Figures 4(f) and 4(g)). In addition, 183 DEGs were screened between DEG4 and MI_1w, which were mainly enriched in neutrophil degranulation, inflammatory response, regulation of cytokine production, and myeloid leukocyte activation (Figures 4(c) and 4(e); Table 4). Further PPI network analysis demonstrated that six modules were related to the pathways, such as Ccl3, Ccr5, Il10ra, Ncf4, and Socs3 (Figures 4(h) and 4(i)).

**Figure 5:**
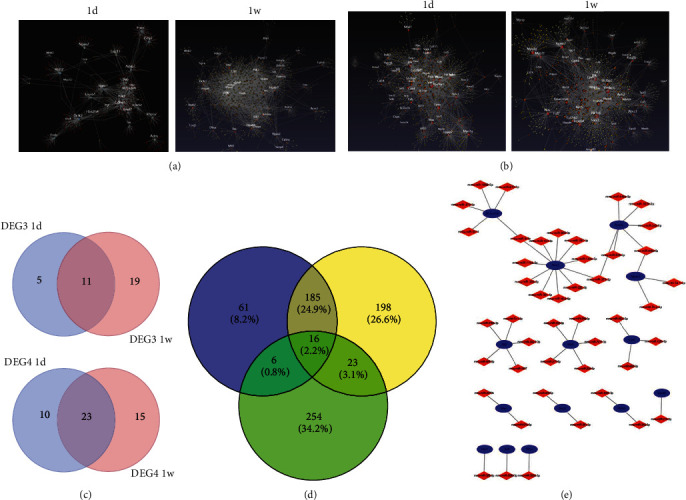
miRNA-hub gene network. (a, b) The screened gene clusters about DEG3 (a) and DEG4 (b) were uploaded to NetworkAnalyst 3.0 for further verification. (c) 11 DEGs and 23 DEGs were obtained at 1 d and 1 w after MI, which may play a critical role in MI recurrence and plaque progression. (d) The different expression miRNA microarray data (1 d and 1 w) in GSE114695 were used to verify the prediction of miRNA. (e) The network of screened hub genes and miRNAs after validation.

The screened gene clusters of DEG3 and DEG4 were then uploaded to NetworkAnalyst 3.0, for further verification (Figures 5(a) and 5(b)).

### 3.4. Further miRNA Mining and Interaction Network Analysis

To investigate the key genes that promote atherosclerosis and MI progression, 11 DEGs in aged mice and 23 DEGs in young mice were selected (Figure 5(c)). After selection, predictions were the best for the 3′UTR of the target gene binding region among the screened hub genes (Figure S6). The miRNA microarray data in GSE114695 were used to verify the prediction of miRNA, and Cytoscape was used to draw the interaction network (Figures 5(d) and 5(e)).

### 3.5. Validations of Screened Hub Genes

**Figure 6:**
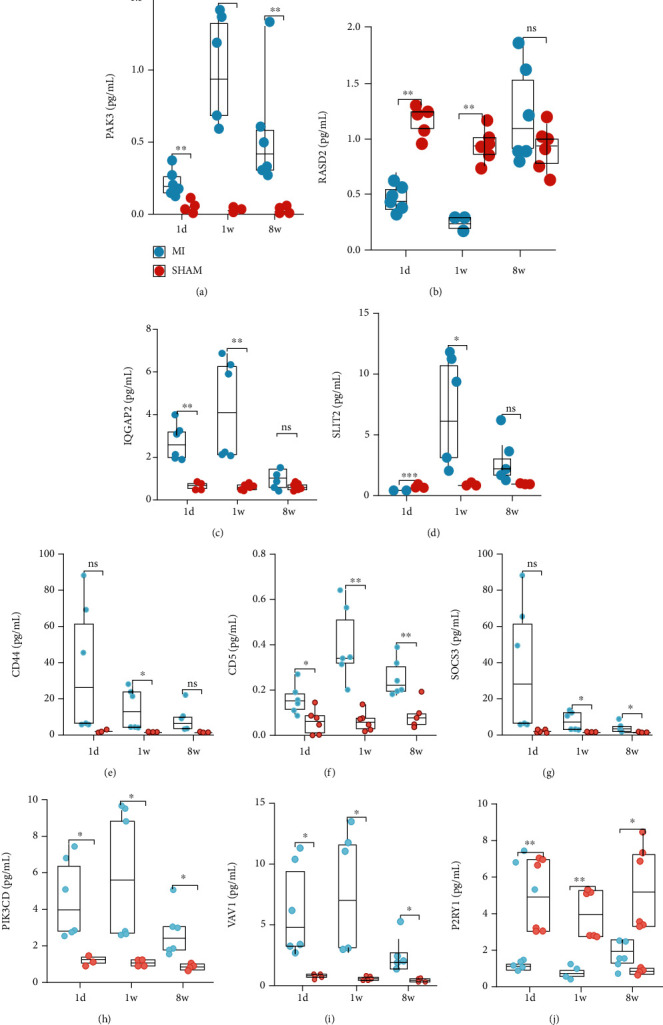
Validations of screened hub genes. The protein expression levels of left ventricle at 1 d, 1 w, and 8 w after MI utilizing ELISA, including PAK3 (a), RASD2 (b), IQGAP2 (c), SLIT2 (d), CD44 (e), CD5 (f), SOCS3 (g), PIK3CD (h), VAV1 (i), and P2RY1 (j).

To validate the function of the screened hub genes in the miRNA-mRNA network, ELISA of the left ventricle was performed in young mice fed with HFD (Figure 6). Expression of PAK3, IQGAP2, CD5, PIK3CD, and VAV1 was upregulated 1 d and 1 w after MI compared with that in the sham group, while there was no significant difference in the expression of CD44 and SOCS3 at 1 d but was highly expressed at 1 wk. In addition, the expression of RASD2, SLIT2, and P2RY1 was lower at 1 d and 1 wk after MI compared with that in the sham group.

**Figure 7:**
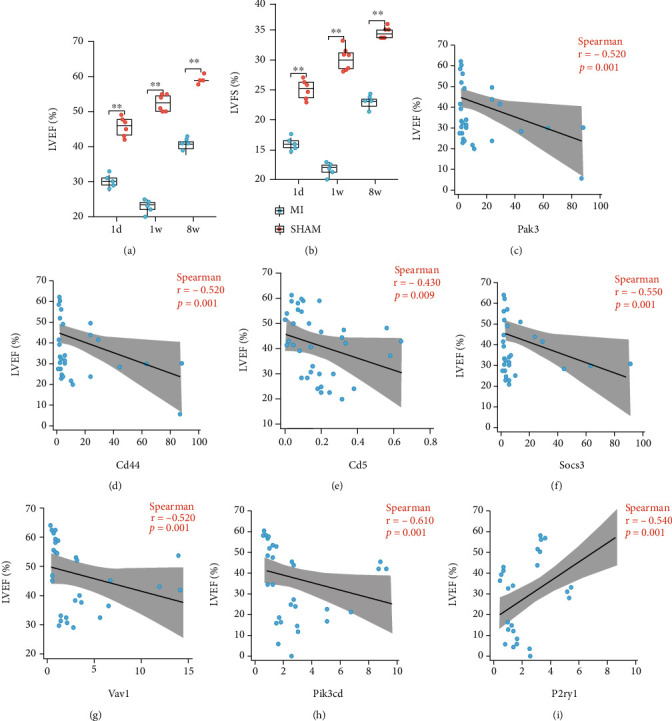
The correlation analysis of screened hub genes and LVEF. (a, b) The LVEF (a) and LVFS (b) of left ventricle at 1 d, 1 w, and 8 w after MI. (c–h) The expression levels of proteins, including PAK3 (c), CD44 (d), CD5 (e), SOCS3 (f), VAV1 (g), and PIK3CD (h) were demonstrated to be negatively correlated with LVEF. (i) The expression levels of protein P2RY1 were demonstrated to be positively correlated with LVEF.

Correlation analysis was used to investigate the cardiac function of the hub genes mentioned above. The expression of proteins, including PAK3, CD44, CD5, SOCS3, VAV1, and PIK3CD, was negatively correlated with LVEF; however, expression of P2RY1 was positively correlated with LVEF (Figure 7). The expression of RASD2, SLIT2, and IQGAP2 was not correlated with cardiac function (Figure S7).

## 4. Discussion

Previous studies have demonstrated the etiology of stable CAD and MI; however, predictive biomarkers and treatment targets are still limited [5, 12]. Previous studies demonstrated that CAD progression, including healthy subjects to stable CAD and stable CAD to STEMI, can induce plaque progression, which may deteriorate CAD progression [6–8]. In the present study, two GEO datasets were utilized, and further analysis, for instance, GO/KEGG enrichment analysis and PPI analysis, was applied to investigate the same DEGs between MI and plaque progression, which may be the critical hub genes that promote MI recurrence and plaque progression.

There is aorta microarray data of young and aged LDLr-deficient mice fed with chow or HFD in GSE69187, while there are left ventricular microarray data at 1 d or 1 or 8 wk subsequent to MI in GSE114695. The same DEGs were screened using a Venn diagram, and further analysis to identify hub genes was applied. The number of the same DEGs between MI and aged vs. young fed with HFD was less than that of the same DEGs between MI and aged versus young fed chow, which may be owing to the higher expression level of adipocytokines and the crosstalk between white adipose tissues and infarcted myocardium [13, 14]. Using the Venn diagram, the DEGs named DEG3 were screened in aged mice fed with HFD compared to those fed with chow, and the DEGs named DEG4 were screened in young mice fed with HFD compared to those fed chow. The same DEGs between MI and DEG4 were used to investigate why young patients with type 2 diabetes and MI have higher long-term all-cause and cardiovascular mortality, and mildly abnormal baseline lipid levels, and not measures of lipid variability, are associated with an increased future risk of atherosclerotic cardiovascular disease events, particularly MI [3, 4].

Ozcebe et al. [15] reported that the adult cardiac extracellular matrix (ECM) improved cardiac function, while aged ECM accelerated the aging phenotype and impaired cardiac function and stress defense machinery of the cells. However, young patients with type 2 diabetes and MI have higher long-term all-cause and cardiovascular mortality, which may be due to the HFD diet. Therefore, we constructed MI mice fed with HFD and validated the cardiac function of the screened hub genes. The hub genes were screened, and miRNA-hub gene networks were also shown after validation.

Seven hub genes were correlated with cardiac function after MI in young mice fed with HFD. P21-activated kinase 3 (PAK3), known to control synaptic plasticity and dendritic spine dynamics, can interact with the Rho guanosine triphosphatase RhoJ. RhoJ acts in opposition to Cdc42 in this process through competition for a shared partner, PAK3, which identifies a critical role for RhoJ in matrix remodeling during blood vessel formation and restricting fibronectin remodeling [16, 17]. CD44, a hyaluronan receptor, can affect crosstalk between fibroblasts and macrophages after MI because cardiac fibroblasts are activated by monocytes/macrophages and, in turn, protect macrophages from apoptosis [18]. Blocking IL-6 decreases ECM formation and LVEF through hyaluronan/CD44 signaling [19]. CD5+ B cells, surface phenotype of IL-10-producing B cells, were enriched in pericardial adipose tissues and accumulated in the infarcted heart during the resolution of MI-induced inflammation [20]. CD5+ B cell-specific deletion can worsen cardiac function, exacerbate myocardial injury, and delay resolution of inflammation following acute MI [20, 21]. Myeloid suppressor of cytokine signaling 3 (SOCS3) expression is an independent risk factor for STEMI and can result in enhanced inflammatory responses; for example, SOCS3 regulates the STAT3/adiponectin axis in response to cardiac injury [22, 23]. In Ldlr-/- mice fed a Western diet, platelets drive atherogenesis by skewing macrophages to an inflammatory phenotype, increasing SOCS3 expression and reducing the Socs1 : Socs3 ratio, thereby promoting inflammatory cytokine production, such as IL-6, IL-1b, and TNF-a [24]. Vav1+ hematopoietic cells (HC) contribute to the developing epicardium, which is not derived from the proepicardial organ [25]. Conditional knockout of Prox1 in Vav1+ compartments revealed that myocardial infarction could promote a significant lymphangiogenic response to improve cardiac function through Vav1+HC and VEGF-C [26]. In addition, a Vav1+ subpopulation of cells is elevated during the first 24 wk of adult life but depleted in aged mice. PIK3CD and P2RY1 have not been reported in MI and atherosclerosis; however, Pik3cd is a critical gene in adipose-derived stem cells, which is the response to inflammation, which may be referred to in studies on diabetes [27]. P2RY1 in supporting cells regulates hair cell excitability by controlling the volume of the extracellular space through nonsynaptic transmission and cross-depolarization [28, 29]. Thus, PIK3CD (PIK3CD/Akt axis) and P2RY1 may be novel potential biomarkers for MI recurrence and plaque progression. Slit2 is a cell motility modulator and a powerful negative regulator of platelet function and thrombus formation [30]. The cytokines tested by ELISA may be the critical crosstalk between the myocardium and other cells, such as macrophages and adipocytes.

Diabetes is also related to oxidative stress changes, apoptosis, and mitochondrial dysfunction [31, 32]. Young patients with type 2 diabetes and MI have higher long-term all-cause and cardiovascular mortality, and more than one-third of patients die within 10 years, which may emphasize more aggressive secondary prevention for these patients [3]. The European Society of Cardiology (ESC) algorithms can be used to rule out or rule MI without ST-elevation in patients with diabetes [33]. In the IMPROVE-IT clinical trial, the benefit of adding ezetimibe to statins was enhanced in patients with diabetes and in high-risk patients without diabetes [34]. The mortality and morbidity of patients with diabetes and those undergoing MI are still increasing, and the increased in-hospital mortality and morbidity of diabetes patients with STEMI are mainly driven by their underlying cardiorenal dysfunction [3, 35]. Although therapies are helpful, patients with diabetes remain a high-risk population for whom identification of MI is challenging and requires careful clinical evaluation. The results in our study may be referred to the researches for patients with diabetes and undergoing MI, thus decreasing mortality and morbidity.

There are some limitations to this study. First, only hub genes were validated for MI progression, and miRNAs were validated through the Venn diagram of prediction of hub genes and miRNA microarray data in GSE114695. There may be some false negatives due to the enrichment and validation methods. More research is still needed to proceed with integrated bioinformatic analysis of miRNAs and their targets in MI recurrence and plaque progression. Second, the sample sizes of the included datasets were not too large; however, after validation, the results were highly reliable. Lastly, further research is needed to confirm the functional effects of screened hub genes on MI recurrence in humans to improve prognosis and decrease MI mortality.

## 5. Conclusion

Based on our current study, our research provided a bioinformatic analysis of MI recurrence and plaque progression, especially in young MI patients with HFD or even diabetes. The screened hub genes, PAK3, CD44, CD5, SOCS3, VAV1, PIK3CD, and P2RY1, may be therapeutic targets for the treatment of STEMI patients and prevention of MI recurrence. PAK3, CD44, CD5, SOCS3, VAV1, and PIK3CD are negatively correlated with cardiac systolic function, and P2RY1 is positively correlated with LVEF, which may be referred to in studies on type 2 diabetes.

## Figures and Tables

**Table 1:** Top clusters with their representative enriched terms of gene lists between DEG3 and MI_1d.

GO	Category	Description	Count	%	log10(*P*)
GO:0001503	GO biological processes	Ossification	8	10.39	-3.94
GO:0030029	GO biological processes	Actin filament-based process	10	12.99	-3.15
GO:0090066	GO biological processes	Regulation of anatomical structure size	8	10.39	-2.83
GO:0060562	GO biological processes	Epithelial tube morphogenesis	6	7.79	-2.47
GO:0019730	GO biological processes	Antimicrobial humoral response	4	5.19	-2.46
GO:0043525	GO biological processes	Positive regulation of neuron apoptotic process	3	3.90	-2.46
GO:0097581	GO biological processes	Lamellipodium organization	3	3.90	-2.38
GO:0006643	GO biological processes	Membrane lipid metabolic process	4	5.19	-2.37
GO:0031532	GO biological processes	Actin cytoskeleton reorganization	3	3.90	-2.12
GO:0001666	GO biological processes	Response to hypoxia	4	5.19	-2.11

“Count” is the number of genes in the user-provided lists with membership in the given ontology term. “%” is the percentage of all of the user-provided genes that are found in the given ontology term (only input genes with at least one ontology term annotation are included in the calculation). “log10(*P*)” is the *P* value in log base 10.

**Table 2:** Top clusters with their representative enriched terms of gene lists between DEG3 and MI_1w.

GO	Category	Description	Count	%	log10(*P*)
R-MMU-194315	Reactome gene sets	Signaling by Rho GTPases	11	9.24	-5.46
GO:0022612	GO biological processes	Gland morphogenesis	7	5.88	-4.49
R-MMU-2682334	Reactome gene sets	EPH-Ephrin signaling	5	4.20	-4.43
GO:0045598	GO biological processes	Regulation of fat cell differentiation	6	5.04	-3.67
GO:0090288	GO biological processes	Negative regulation of cellular response to growth factor stimulus	6	5.04	-3.59
GO:0033555	GO biological processes	Multicellular organismal response to stress	5	4.20	-3.52
R-MMU-418592	Reactome gene sets	ADP signaling through P2Y purinoceptor 1	3	2.52	-3.46
GO:0050995	GO biological processes	Negative regulation of lipid catabolic process	3	2.52	-3.40
R-MMU-9033241	Reactome gene sets	Peroxisomal protein import	4	3.36	-3.31
GO:1903010	GO biological processes	Regulation of bone development	3	2.52	-3.30

“Count” is the number of genes in the user-provided lists with membership in the given ontology term. “%” is the percentage of all of the user-provided genes that are found in the given ontology term (only input genes with at least one ontology term annotation are included in the calculation). “log10(*P*)” is the *P* value in log base 10.

**Table 3:** Top clusters with their representative enriched terms of gene lists between DEG4 and MI_1d.

GO	Category	Description	Count	%	log10(*P*)
GO:0006954	GO biological processes	Inflammatory response	27	19.01	-11.96
GO:0042110	GO biological processes	T cell activation	21	14.79	-10.16
GO:0002274	GO biological processes	Myeloid leukocyte activation	15	10.56	-10.10
R-MMU-6798695	Reactome gene sets	Neutrophil degranulation	20	14.08	-9.38
GO:0002250	GO biological processes	Adaptive immune response	20	14.08	-8.35
GO:0019221	GO biological processes	Cytokine-mediated signaling pathway	16	11.27	-8.22
GO:0060326	GO biological processes	Cell chemotaxis	14	9.86	-7.58
GO:0001906	GO biological processes	Cell killing	11	7.75	-6.83
GO:0002683	GO biological processes	Negative regulation of immune system process	16	11.27	-6.60
R-MMU-983695	Reactome gene sets	Antigen activates B cell receptor (BCR) leading to generation of second messengers	5	3.52	-6.35
GO:0002366	GO biological processes	Leukocyte activation involved in immune response	12	8.45	-6.14
ko04668	KEGG pathway	TNF signaling pathway	8	5.63	-6.11
GO:2000106	GO biological processes	Regulation of leukocyte apoptotic process	8	5.63	-6.02
GO:0002526	GO biological processes	Acute inflammatory response	8	5.63	-5.65
ko05152	KEGG pathway	Tuberculosis	9	6.34	-5.38
GO:0006909	GO biological processes	Phagocytosis	12	8.45	-5.13
R-MMU-109582	Reactome gene sets	Hemostasis	14	9.86	-4.96
GO:0033628	GO biological processes	Regulation of cell adhesion mediated by integrin	5	3.52	-4.89
GO:0031334	GO biological processes	Positive regulation of protein complex assembly	10	7.04	-4.67
GO:0009620	GO biological processes	Response to fungus	5	3.52	-4.53

“Count” is the number of genes in the user-provided lists with membership in the given ontology term. “%” is the percentage of all of the user-provided genes that are found in the given ontology term (only input genes with at least one ontology term annotation are included in the calculation). “log10(*P*)” is the *P* value in log base 10.

**Table 4:** Top clusters with their representative enriched terms of gene lists between DEG4 and MI_1w.

GO	Category	Description	Count	%	log10(*P*)
R-MMU-6798695	Reactome gene sets	Neutrophil degranulation	24	13.41	-10.67
GO:0006954	GO biological processes	Inflammatory response	28	15.64	-10.25
GO:0001817	GO biological processes	Regulation of cytokine production	27	15.08	-9.68
GO:0002274	GO biological processes	Myeloid leukocyte activation	15	8.38	-8.68
GO:0002683	GO biological processes	Negative regulation of immune system process	20	11.17	-8.04
GO:0002250	GO biological processes	Adaptive immune response	22	12.29	-7.99
GO:0071216	GO biological processes	Cellular response to biotic stimulus	15	8.38	-6.84
ko04662	KEGG pathway	B cell receptor signaling pathway	8	4.47	-6.68
ko05150	KEGG pathway	Staphylococcus aureus infection	7	3.91	-6.49
GO:0050778	GO biological processes	Positive regulation of immune response	22	12.29	-5.94
mmu04640	KEGG pathway	Hematopoietic cell lineage	8	4.47	-5.54
GO:0006925	GO biological processes	Inflammatory cell apoptotic process	5	2.79	-5.49
GO:0050900	GO biological processes	Leukocyte migration	14	7.82	-5.46
ko04380	KEGG pathway	Osteoclast differentiation	8	4.47	-4.73
GO:0030050	GO biological processes	Vesicle transport along actin filament	4	2.23	-4.71
ko04620	KEGG pathway	Toll-like receptor signaling pathway	7	3.91	-4.59
GO:0033627	GO biological processes	Cell adhesion mediated by integrin	6	3.35	-4.52
GO:0045453	GO biological processes	Bone resorption	6	3.35	-4.21
GO:0002521	GO biological processes	Leukocyte differentiation	16	8.94	-4.21
GO:0030029	GO biological processes	Actin filament-based process	19	10.61	-4.20

“Count” is the number of genes in the user-provided lists with membership in the given ontology term. “%” is the percentage of all of the user-provided genes that are found in the given ontology term (only input genes with at least one ontology term annotation are included in the calculation). “log10(*P*)” is the *P* value in log base 10.

## Data Availability

The datasets can be found in GEO datasets, NCBI.

## Supplementary Materials

Supplementary 1 Supplementary Figure 1: DEGs of left ventricular samples between MI and sham in GSE114695 after log2 transformation. Bar plot and principal component analysis of DEGs at 1 d (A), 1 w (B), and 8 w (C) after MI.

Supplementary 2 Supplementary Figure 2: DEGs of plaque samples in GSE69187 after log2 transformation. (A) Bar plot and principal component analysis of DEGs of plaque samples between aged mice fed with chow and aged mice fed with HFD. (B) Bar plot and principal component analysis of DEGs of plaque samples between young mice fed with chow and young mice fed with HFD. (C) Bar plot and principal component analysis of DEGs of plaque samples between aged mice and young mice fed with chow. (D) Bar plot and principal component analysis of DEGs of plaque samples between aged mice and young mice fed with HFD.

Supplementary 3 Supplementary Figure 3: enrichment analysis of DEGs at 1 w and 8 w in GSE114695. (A) 763 DEGs were upregulated at 1 w and 8 w after MI, and enrichment analysis demonstrated DEGs were mainly enriched in inflammatory response, cell-cell adhesion, and extracellular matrix organization. (B) 507 DEGs were both downregulated at 1 w and 8 w after MI, which were mainly enriched in regulation of ion transmembrane transport, regulation of membrane potential, and action potential. (C) 9 DEGs were different between 1 w and 8 w, which were enriched in positive regulation of cell morphogenesis involved in differentiation, glial cell differentiation, and regulation of neuron differentiation.

Supplementary 4 Supplementary Figure 4: enrichment analysis of DEGs at 1 d in GSE114695. (A) Venn diagram of DEG1 and DEGs at 1 d demonstrated 2360 DEGs were in DEGs at 1 d but not in DEG1. (B) Enrichment analysis showed 2360 DEGs were mainly enriched in inflammatory response, leukocyte migration, regulation of cytokine production, and T cell activation. (C) Network of enriched pathways. (D-E) PPI of 2360 DEGs (D) and module in PPI (E).

Supplementary 5 Supplementary Figure 5: enrichment analysis of DEGs at 1 w in GSE114695. (A) Venn diagram of DEG1 and DEGs at 1 w demonstrated 3171 DEGs were in DEGs at 1 w but not in DEG1. (B) Enrichment analysis showed 3171 DEGs were mainly enriched in cell-cell adhesion, inflammatory response, and regulation of ion transport. (C) Network of enriched pathways.

Supplementary 6 Supplementary Figure 6: the selection of the target gene binding region. (A-C) Venn diagram between miRNAs which bind the 5′UTR, CDS, and 3′UTR of hub genes and 1 d (A), 1 w (B), and 8 w (C), demonstrating that the target gene binding region 3′UTR was the best predicted binding region of screened hub genes. (D) Venn diagram of the different expression miRNA microarray data (1 d, 1 w, and 8 w) and miRNAs which bind the 3′UTR of hub genes.

Supplementary 7 Supplementary Figure 7: the correlation analysis of screened hub genes and LVEF. The expression levels of RASD2 (A), SLIT2 (B), and IQGAP2 (C) were not correlated with cardiac function.

Supplementary 8 Supplementary Table 1: top clusters with their representative enriched terms of gene lists between MI_1d up and MI_1w up. Supplementary Table 2: top clusters with their representative enriched terms of gene lists between MI_1d down and MI_1w down. Supplementary Table 3: top clusters with their representative enriched terms of gene lists between MI_1w up and MI_8w up. Supplementary Table 4: Top clusters with their representative enriched terms of gene lists between MI_1w down and MI_8w down.
