# Supplementary figures and images for: The Key Genes Underlying Pathophysiology Association between Plaque Instability and Progression of Myocardial Infarction
Source: Dis Markers. 2021 Dec 9;2021:4300406. doi: 10.1155/2021/4300406 (PMC8678557; doi:10.1155/2021/4300406)

A

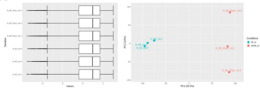

B

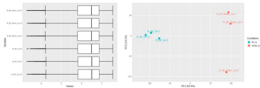

C

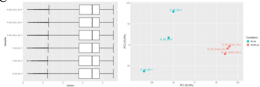

Supplement: Supplementary 1 — Supplementary Figure 1: DEGs of left ventricular samples between MI and sham in GSE114695 after log2 transformation. Bar plot and principal component analysis of DEGs at 1 d (A), 1 w (B), and 8 w (C) after MI. [file 4300406.f1.pdf]

A

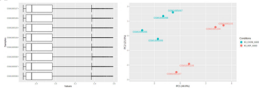

B

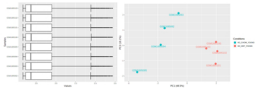

C

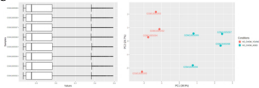

D

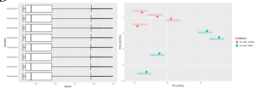

Supplement: Supplementary 2 — Supplementary Figure 2: DEGs of plaque samples in GSE69187 after log2 transformation. (A) Bar plot and principal component analysis of DEGs of plaque samples between aged mice fed with chow and aged mice fed with HFD. (B) Bar plot and principal component analysis of DEGs of plaque samples between young mice fed with chow and young mice fed with HFD. (C) Bar plot and principal component analysis of DEGs of plaque samples between aged mice and young mice fed with chow. (D) Bar plot and principal component analysis of DEGs of plaque samples between aged mice and young mice fed with HFD. [file 4300406.f2.pdf]

A

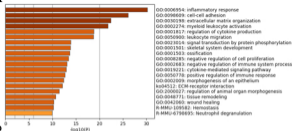

B

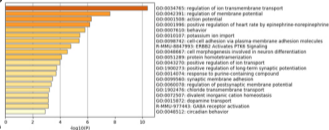

C

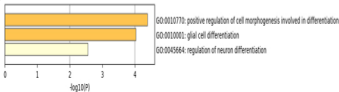

Supplement: Supplementary 3 — Supplementary Figure 3: enrichment analysis of DEGs at 1 w and 8 w in GSE114695. (A) 763 DEGs were upregulated at 1 w and 8 w after MI, and enrichment analysis demonstrated DEGs were mainly enriched in inflammatory response, cell-cell adhesion, and extracellular matrix organization. (B) 507 DEGs were both downregulated at 1 w and 8 w after MI, which were mainly enriched in regulation of ion transmembrane transport, regulation of membrane potential, and action potential. (C) 9 DEGs were different between 1 w and 8 w, which were enriched in positive regulation of cell morphogenesis involved in differentiation, glial cell differentiation, and regulation of neuron differentiation. [file 4300406.f3.pdf]

A

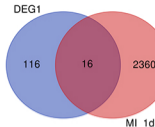

B

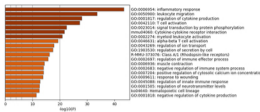

C

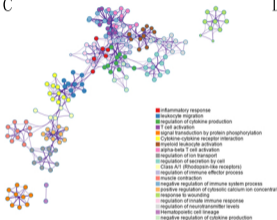

D

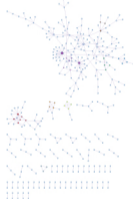

E

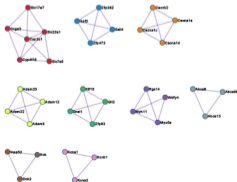

Supplement: Supplementary 4 — Supplementary Figure 4: enrichment analysis of DEGs at 1 d in GSE114695. (A) Venn diagram of DEG1 and DEGs at 1 d demonstrated 2360 DEGs were in DEGs at 1 d but not in DEG1. (B) Enrichment analysis showed 2360 DEGs were mainly enriched in inflammatory response, leukocyte migration, regulation of cytokine production, and T cell activation. (C) Network of enriched pathways. (D-E) PPI of 2360 DEGs (D) and module in PPI (E). [file 4300406.f4.pdf]

A

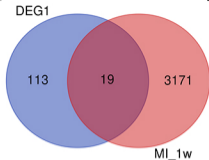

B

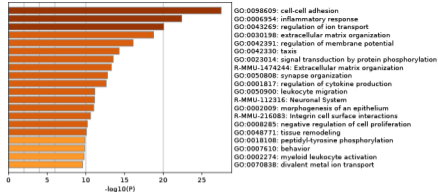

C

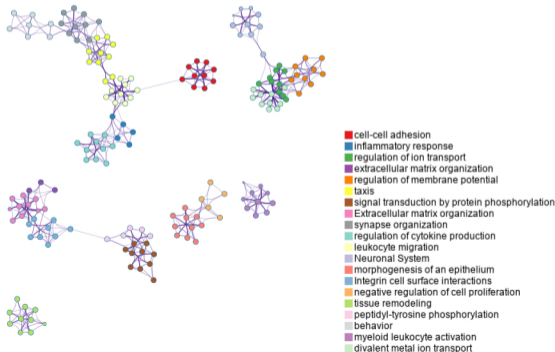

Supplement: Supplementary 5 — Supplementary Figure 5: enrichment analysis of DEGs at 1 w in GSE114695. (A) Venn diagram of DEG1 and DEGs at 1 w demonstrated 3171 DEGs were in DEGs at 1 w but not in DEG1. (B) Enrichment analysis showed 3171 DEGs were mainly enriched in cell-cell adhesion, inflammatory response, and regulation of ion transport. (C) Network of enriched pathways. [file 4300406.f5.pdf]

A

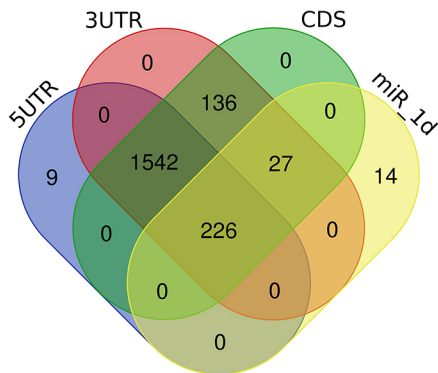

B

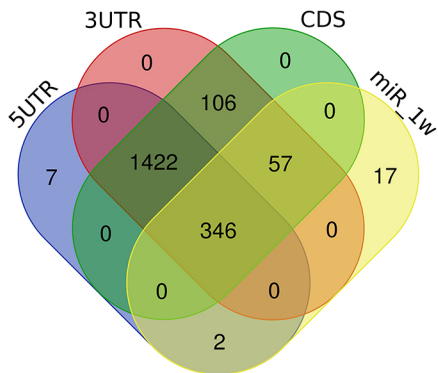

C

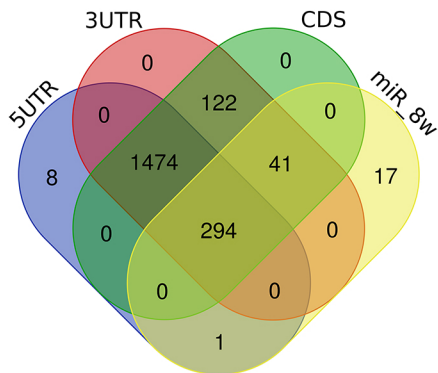

D

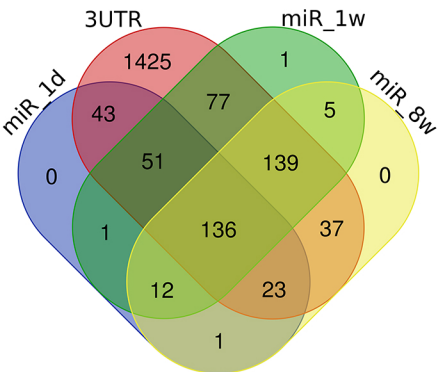

Supplement: Supplementary 6 — Supplementary Figure 6: the selection of the target gene binding region. (A-C) Venn diagram between miRNAs which bind the 5′UTR, CDS, and 3′UTR of hub genes and 1 d (A), 1 w (B), and 8 w (C), demonstrating that the target gene binding region 3′UTR was the best predicted binding region of screened hub genes. (D) Venn diagram of the different expression miRNA microarray data (1 d, 1 w, and 8 w) and miRNAs which bind the 3′UTR of hub genes. [file 4300406.f6.pdf]

**A**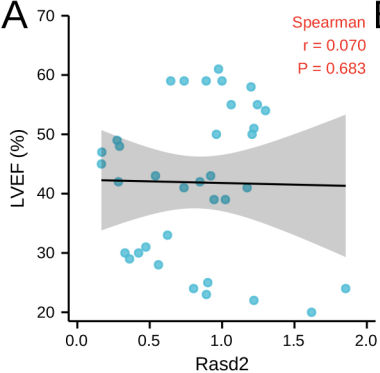**B**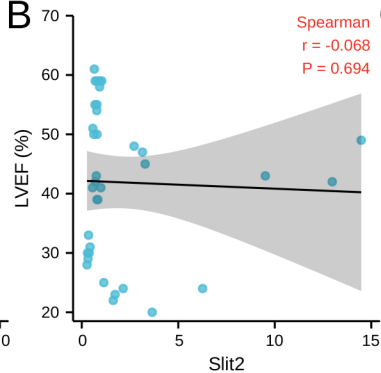**C**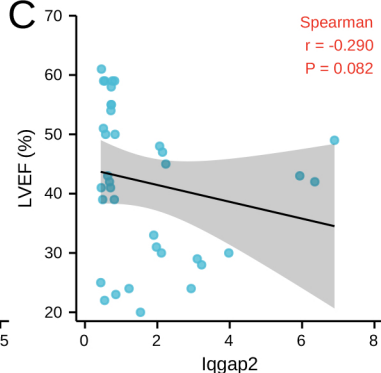

Supplement: Supplementary 7 — Supplementary Figure 7: the correlation analysis of screened hub genes and LVEF. The expression levels of RASD2 (A), SLIT2 (B), and IQGAP2 (C) were not correlated with cardiac function. [file 4300406.f7.pdf]
