# Supplementary material for: The Key Genes Underlying Pathophysiology Association between Plaque Instability and Progression of Myocardial Infarction
Source: Dis Markers. 2021 Dec 9;2021:4300406. doi: 10.1155/2021/4300406 (PMC8678557; doi:10.1155/2021/4300406)
Supplement: Supplementary 8 — Supplementary Table 1: top clusters with their representative enriched terms of gene lists between MI_1d up and MI_1w up. Supplementary Table 2: top clusters with their representative enriched terms of gene lists between MI_1d down and MI_1w down. Supplementary Table 3: top clusters with their representative enriched terms of gene lists between MI_1w up and MI_8w up. Supplementary Table 4: Top clusters with their representative enriched terms of gene lists between MI_1w down and MI_8w down. [file 4300406.f8.doc]

Supplementary Table 1. Top clusters with their representative enriched terms of gene lists between MI_1d Up and MI_1w Up.

| **GO** | **Category** | **Description** | **Count** | **%** | **Log10(P)** |
| --- | --- | --- | --- | --- | --- |
| GO:0006954 | GO Biological Processes | inflammatory response | 118 | 18.41 | -50.00 |
| GO:0002274 | GO Biological Processes | myeloid leukocyte activation | 56 | 8.74 | -32.99 |
| GO:0050900 | GO Biological Processes | leukocyte migration | 67 | 10.45 | -32.14 |
| GO:0042110 | GO Biological Processes | T cell activation | 77 | 12.01 | -30.05 |
| GO:0050778 | GO Biological Processes | positive regulation of immune response | 87 | 13.57 | -24.93 |
| GO:0002683 | GO Biological Processes | negative regulation of immune system process | 64 | 9.98 | -22.10 |
| GO:0032635 | GO Biological Processes | interleukin-6 production | 36 | 5.62 | -19.53 |
| GO:0002697 | GO Biological Processes | regulation of immune effector process | 57 | 8.89 | -19.27 |
| GO:0019221 | GO Biological Processes | cytokine-mediated signaling pathway | 52 | 8.11 | -19.18 |
| GO:1903555 | GO Biological Processes | regulation of tumor necrosis factor superfamily cytokine production | 34 | 5.30 | -17.23 |
| GO:0098542 | GO Biological Processes | defense response to other organism | 71 | 11.08 | -17.22 |
| R-MMU-6798695 | Reactome Gene Sets | Neutrophil degranulation | 58 | 9.05 | -16.73 |
| mmu04640 | KEGG Pathway | Hematopoietic cell lineage | 26 | 4.06 | -16.35 |
| GO:0023014 | GO Biological Processes | signal transduction by protein phosphorylation | 71 | 11.08 | -15.23 |
| R-MMU-109582 | Reactome Gene Sets | Hemostasis | 54 | 8.42 | -14.59 |
| GO:0032609 | GO Biological Processes | interferon-gamma production | 27 | 4.21 | -14.52 |
| GO:0001776 | GO Biological Processes | leukocyte homeostasis | 24 | 3.74 | -12.94 |
| GO:0001818 | GO Biological Processes | negative regulation of cytokine production | 35 | 5.46 | -12.25 |
| ko04650 | KEGG Pathway | Natural killer cell mediated cytotoxicity | 23 | 3.59 | -11.77 |
| ko04380 | KEGG Pathway | Osteoclast differentiation | 24 | 3.74 | -11.72 |

"Count" is the number of genes in the user-provided lists with membership in the given ontology term. "%" is the percentage of all of the user-provided genes that are found in the given ontology term (only input genes with at least one ontology term annotation are included in the calculation). "Log10(P)" is the p-value in log base 10.

Supplementary Table 2. Top clusters with their representative enriched terms of gene lists between MI_1d Down and MI_1w Down.

| **GO** | **Category** | **Description** | **Count** | **%** | **Log10(P)** |
| --- | --- | --- | --- | --- | --- |
| GO:0043269 | GO Biological Processes | regulation of ion transport | 59 | 11.75 | -14.83 |
| GO:0042391 | GO Biological Processes | regulation of membrane potential | 40 | 7.97 | -11.65 |
| GO:0001505 | GO Biological Processes | regulation of neurotransmitter levels | 30 | 5.98 | -7.66 |
| GO:0007610 | GO Biological Processes | behavior | 44 | 8.76 | -7.19 |
| GO:0010107 | GO Biological Processes | potassium ion import | 10 | 1.99 | -6.71 |
| R-MMU-1296072 | Reactome Gene Sets | Voltage gated Potassium channels | 9 | 1.79 | -6.30 |
| GO:0007200 | GO Biological Processes | phospholipase C-activating G protein-coupled receptor signaling pathway | 13 | 2.59 | -6.07 |
| GO:0045823 | GO Biological Processes | positive regulation of heart contraction | 9 | 1.79 | -6.02 |
| R-MMU-5576891 | Reactome Gene Sets | Cardiac conduction | 14 | 2.79 | -5.81 |
| GO:0023061 | GO Biological Processes | signal release | 31 | 6.18 | -5.06 |
| GO:0019228 | GO Biological Processes | neuronal action potential | 8 | 1.59 | -4.86 |
| GO:0007626 | GO Biological Processes | locomotory behavior | 19 | 3.78 | -4.68 |
| GO:0006820 | GO Biological Processes | anion transport | 32 | 6.37 | -4.65 |
| GO:0050803 | GO Biological Processes | regulation of synapse structure or activity | 20 | 3.98 | -4.48 |
| GO:0045913 | GO Biological Processes | positive regulation of carbohydrate metabolic process | 10 | 1.99 | -4.25 |
| R-MMU-209905 | Reactome Gene Sets | Catecholamine biosynthesis | 3 | 0.60 | -4.25 |
| mmu04911 | KEGG Pathway | Insulin secretion | 10 | 1.99 | -4.21 |
| R-MMU-112315 | Reactome Gene Sets | Transmission across Chemical Synapses | 15 | 2.99 | -4.05 |
| GO:0015698 | GO Biological Processes | inorganic anion transport | 12 | 2.39 | -3.65 |
| GO:1903539 | GO Biological Processes | protein localization to postsynaptic membrane | 7 | 1.39 | -3.44 |

"Count" is the number of genes in the user-provided lists with membership in the given ontology term. "%" is the percentage of all of the user-provided genes that are found in the given ontology term (only input genes with at least one ontology term annotation are included in the calculation). "Log10(P)" is the p-value in log base 10.

Supplementary Table 3. Top clusters with their representative enriched terms of gene lists between MI_1w Up and MI_8w Up.

| **GO** | **Category** | **Description** | **Count** | **%** | **Log10(P)** |
| --- | --- | --- | --- | --- | --- |
| GO:0006954 | GO Biological Processes | inflammatory response | 99 | 13.60 | -30.20 |
| GO:0098609 | GO Biological Processes | cell-cell adhesion | 95 | 13.05 | -26.12 |
| GO:0030198 | GO Biological Processes | extracellular matrix organization | 54 | 7.42 | -22.37 |
| GO:0002274 | GO Biological Processes | myeloid leukocyte activation | 47 | 6.46 | -21.81 |
| GO:0001817 | GO Biological Processes | regulation of cytokine production | 80 | 10.99 | -18.83 |
| GO:0050900 | GO Biological Processes | leukocyte migration | 54 | 7.42 | -18.72 |
| GO:0023014 | GO Biological Processes | signal transduction by protein phosphorylation | 74 | 10.16 | -13.88 |
| GO:0001501 | GO Biological Processes | skeletal system development | 58 | 7.97 | -13.83 |
| GO:0001503 | GO Biological Processes | ossification | 49 | 6.73 | -13.67 |
| GO:0008285 | GO Biological Processes | negative regulation of cell proliferation | 68 | 9.34 | -13.40 |
| GO:0002683 | GO Biological Processes | negative regulation of immune system process | 54 | 7.42 | -13.08 |
| GO:0019221 | GO Biological Processes | cytokine-mediated signaling pathway | 46 | 6.32 | -12.90 |
| GO:0050778 | GO Biological Processes | positive regulation of immune response | 71 | 9.75 | -12.66 |
| GO:0002009 | GO Biological Processes | morphogenesis of an epithelium | 58 | 7.97 | -12.36 |
| ko04512 | KEGG Pathway | ECM-receptor interaction | 21 | 2.88 | -12.10 |
| GO:2000027 | GO Biological Processes | regulation of animal organ morphogenesis | 32 | 4.40 | -11.44 |
| GO:0048771 | GO Biological Processes | tissue remodeling | 30 | 4.12 | -10.69 |
| GO:0042060 | GO Biological Processes | wound healing | 43 | 5.91 | -10.24 |
| R-MMU-109582 | Reactome Gene Sets | Hemostasis | 50 | 6.87 | -10.23 |
| R-MMU-6798695 | Reactome Gene Sets | Neutrophil degranulation | 50 | 6.87 | -9.96 |

"Count" is the number of genes in the user-provided lists with membership in the given ontology term. "%" is the percentage of all of the user-provided genes that are found in the given ontology term (only input genes with at least one ontology term annotation are included in the calculation). "Log10(P)" is the p-value in log base 10.

Supplementary Table 4. Top clusters with their representative enriched terms of gene lists between MI_1w Down and MI_8w Down.

| **GO** | **Category** | **Description** | **Count** | **%** | **Log10(P)** |
| --- | --- | --- | --- | --- | --- |
| GO:0034765 | GO Biological Processes | regulation of ion transmembrane transport | 35 | 8.73 | -10.37 |
| GO:0042391 | GO Biological Processes | regulation of membrane potential | 29 | 7.23 | -7.64 |
| GO:0001508 | GO Biological Processes | action potential | 14 | 3.49 | -6.27 |
| GO:0001996 | GO Biological Processes | positive regulation of heart rate by epinephrine-norepinephrine | 4 | 1.00 | -6.15 |
| GO:0007610 | GO Biological Processes | behavior | 35 | 8.73 | -5.80 |
| GO:0010107 | GO Biological Processes | potassium ion import | 8 | 2.00 | -5.43 |
| GO:0098742 | GO Biological Processes | cell-cell adhesion via plasma-membrane adhesion molecules | 16 | 3.99 | -5.38 |
| R-MMU-8847993 | Reactome Gene Sets | ERBB2 Activates PTK6 Signaling | 4 | 1.00 | -4.78 |
| GO:0048667 | GO Biological Processes | cell morphogenesis involved in neuron differentiation | 29 | 7.23 | -4.51 |
| GO:0051289 | GO Biological Processes | protein homotetramerization | 7 | 1.75 | -4.09 |
| GO:0043270 | GO Biological Processes | positive regulation of ion transport | 18 | 4.49 | -3.96 |
| GO:1900273 | GO Biological Processes | positive regulation of long-term synaptic potentiation | 5 | 1.25 | -3.73 |
| GO:0014074 | GO Biological Processes | response to purine-containing compound | 9 | 2.24 | -3.70 |
| GO:0099560 | GO Biological Processes | synaptic membrane adhesion | 5 | 1.25 | -3.44 |
| GO:0060078 | GO Biological Processes | regulation of postsynaptic membrane potential | 9 | 2.24 | -3.34 |
| GO:1902476 | GO Biological Processes | chloride transmembrane transport | 6 | 1.50 | -3.21 |
| GO:0072507 | GO Biological Processes | divalent inorganic cation homeostasis | 22 | 5.49 | -3.13 |
| GO:0015872 | GO Biological Processes | dopamine transport | 6 | 1.50 | -3.12 |
| R-MMU-977443 | Reactome Gene Sets | GABA receptor activation | 6 | 1.50 | -3.12 |
| GO:0048512 | GO Biological Processes | circadian behavior | 6 | 1.50 | -2.89 |

"Count" is the number of genes in the user-provided lists with membership in the given ontology term. "%" is the percentage of all of the user-provided genes that are found in the given ontology term (only input genes with at least one ontology term annotation are included in the calculation). "Log10(P)" is the p-value in log base 10.
